# Supplementary material for: Ocular tropism of SARS-CoV-2 in animal models with retinal inflammation via neuronal invasion following intranasal inoculation
Source: Nat Commun. 2022 Dec 12;13:7675. doi: 10.1038/s41467-022-35225-1 (PMC9743116; doi:10.1038/s41467-022-35225-1)
Supplement: Supplementary file 1 — Supplementary Information [file 41467_2022_35225_MOESM1_ESM.pdf]

Supplementary Information

Ocular tropism of SARS-CoV-2 in animal models with retinal inflammation via neuronal invasion following intranasal inoculation

Jeong et al.

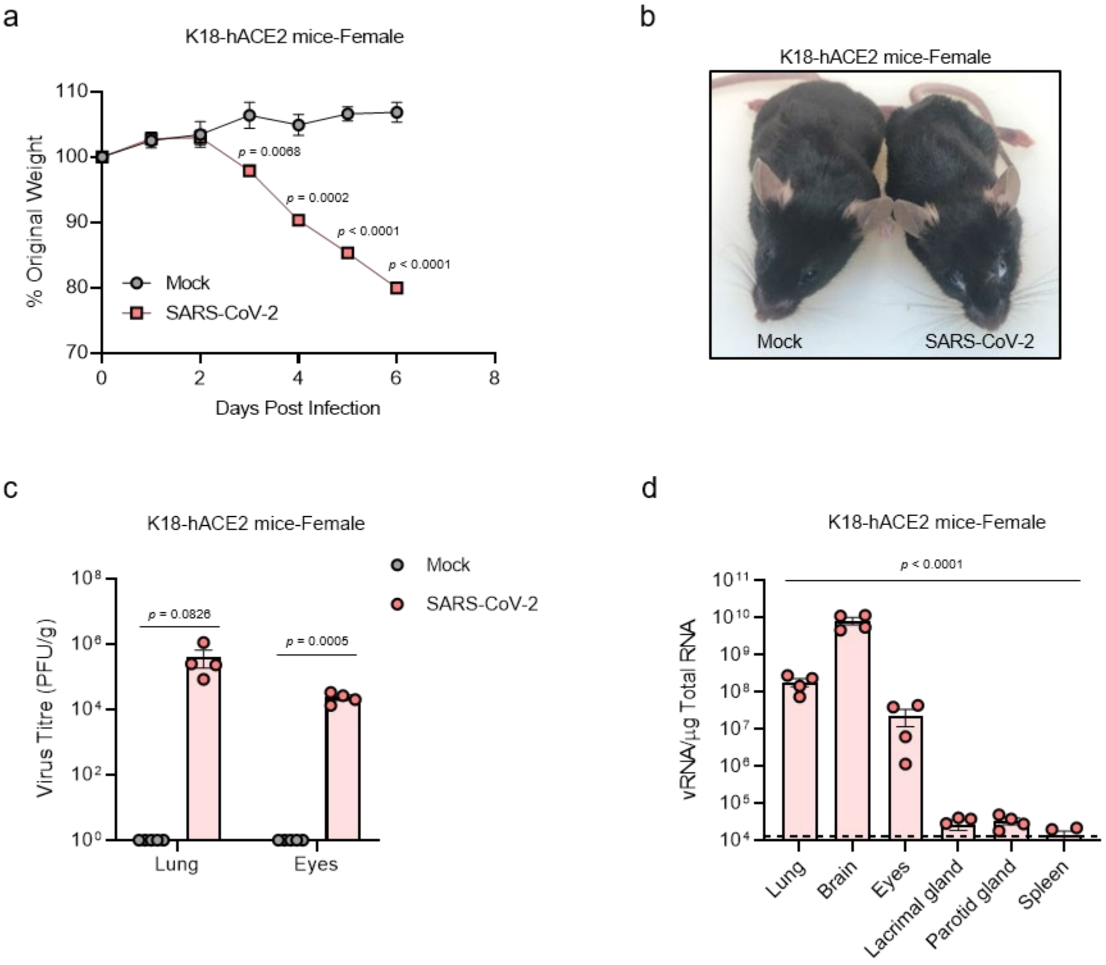

Supplementary Fig. 1. Clinical features and virus titres in the eyes of SARS-CoV-2-infected female mice. Eight-week-old female K18-hACE2 mice were intranasally mock-infected or infected with 10^4 PFU of SARS-CoV-2 ($n = 4$ for mock-infected and infected mice, respectively; mock, gary; SARS-CoV-2, red). **a** Body weight changes shown as percentage of starting weight at the indicated dpi. **b** Representative image of tearing and eye discharge in SARS-CoV-2-infected female mice at 6 dpi (right) compared to that in mock-infected female mice (left). **c** Viral load in the lungs and eyes, including appendages, was analysed using the plaque assay at 6 dpi. **d** Viral RNA levels in the lungs, brain, eyes, including appendages, lacrimal gland, parotid gland, spleen, and colon were assessed using RT-qPCR at 6 dpi. Viral RNA copies were cut-off at 10^4 copies/μg. A dashed line indicates the viral RNA levels of spleen as the limit of detection. Symbols represent means ± SEM. Statistically significant differences between the groups were determined using multiple two-tailed t-tests (**a**), unpaired two-

tailed t-test (**c**), or one-way ANOVA (**d**). SARS-CoV-2: severe acute respiratory syndrome coronavirus 2; PFU: plaque-forming unit; dpi: days post-infection

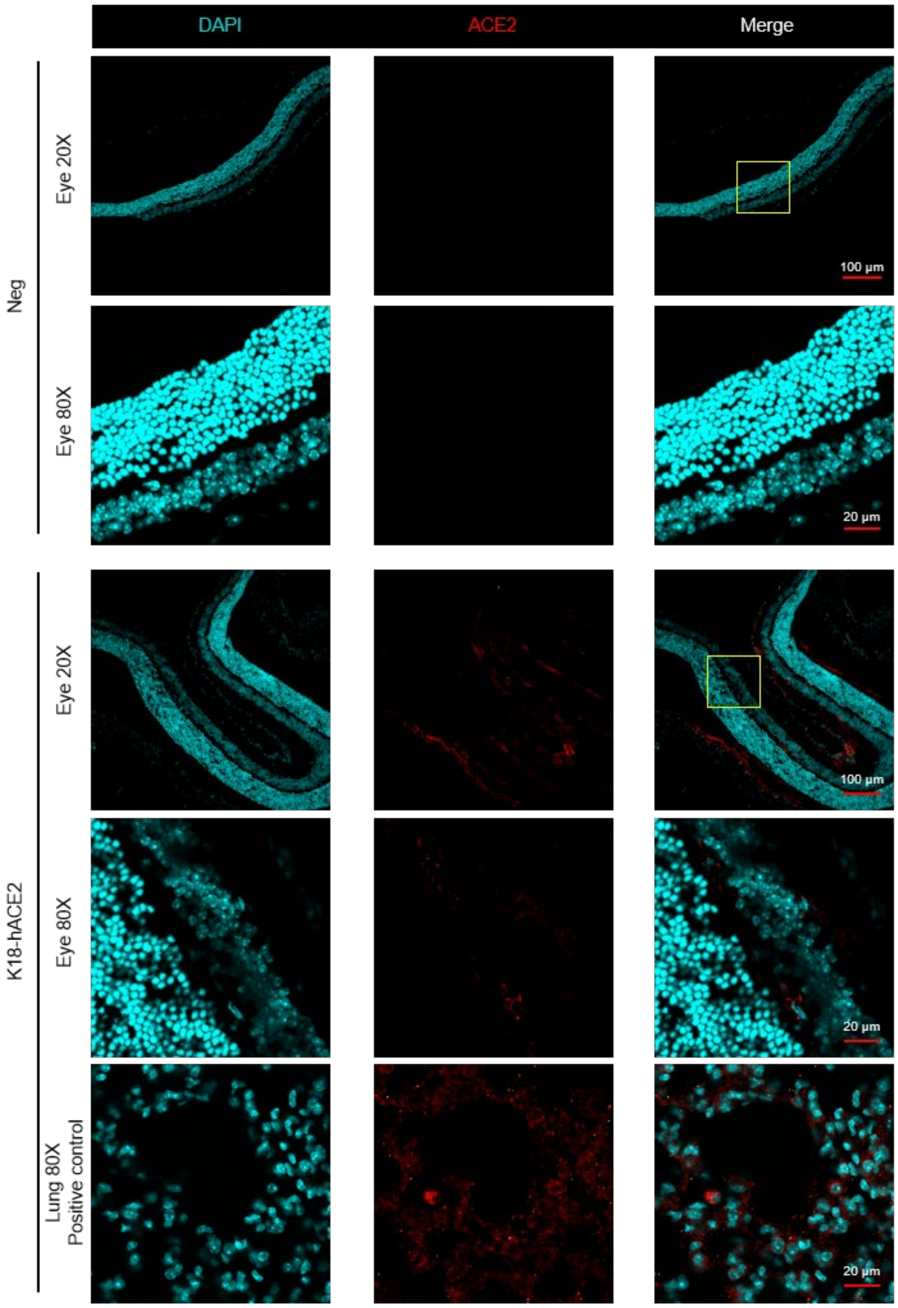

Supplementary Fig. 2. Immunofluorescence staining for hACE2 in the eyes of K18-hACE2 mice. Confocal microscopy of 10 μm cryosections of the eye of C57BL/6 (Neg) or K18-hACE2 mice. Eye and lung tissues were collected and processed for immunofluorescence staining of hACE2. Lung tissue was used as the positive control. Images were acquired using the 10×, 20×, and 40× (with 2× zoom) objectives of a confocal microscope and are representative of *n* = 10 samples. Scale bars in panel = 100 μm for 20× objective and 20 μm for 40× (with 2× zoom) objective. Data are representative of two independent experiments.

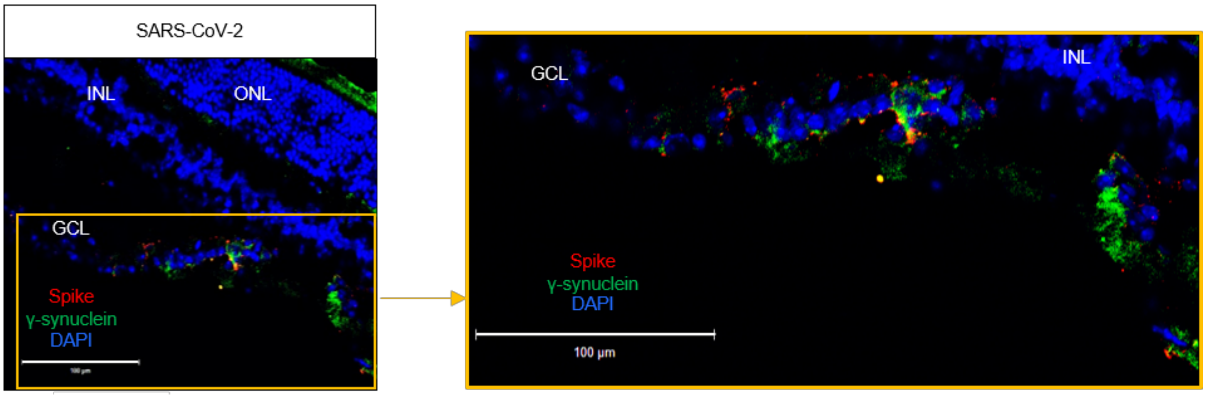

Supplementary Fig. 3. Representative confocal images of fluorescently-stained retinal sections of IN-infected mice for viral spike (S) protein (red) and γ-synuclein (green) at 6 dpi. Eight-week-old female K18-hACE2 mice were intranasally infected with 10^4 PFU of SARS-CoV-2 (n = 3). DAPI staining (blue) was used to visualise nuclei of ganglion cell layer (GCL), inner nuclear layer (INL), and outer nuclear layer (ONL) in the retinal cross section. Scale bar = 100 μm. Data are representative of two independent experiments.

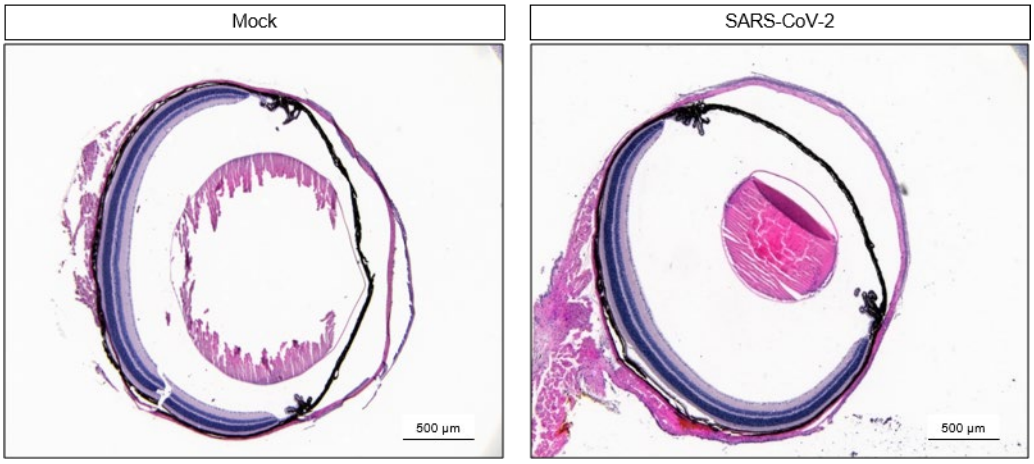

Supplementary Fig. 4. Histopathological analysis of the eyes of SARS-Cov-2-infected mice. H&E staining of the eyeball sections from K18-hACE2 mice (*n* = 4 per group, total eight eyes) six days after mock infection or SARS-CoV-2 infection. Images are representative of total eight eyes. Scale bar = 500 μm.

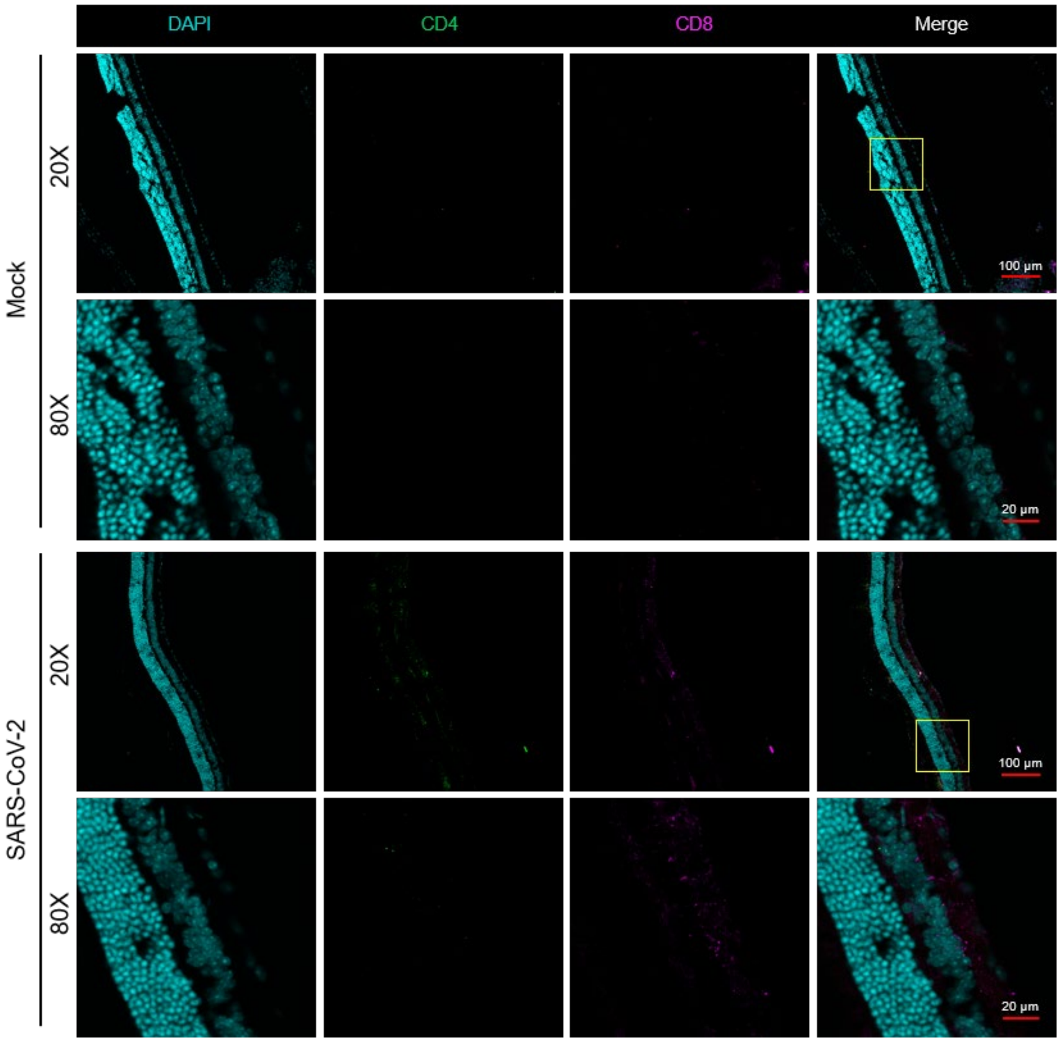

Supplementary Fig. 5. Immunofluorescence staining of immune cells in the eyes of SARS-CoV-2-infected mice. Six to seven-week-old K18-hACE2 mice were infected intranasally with 10^4 PFU SARS-CoV-2 (n = 10) or mock infected with PBS (n = 8). At 6 dpi, eye tissues were collected and processed for immunofluorescence staining. Cryosections were labelled for CD4 and CD8 T cells. Images were acquired using 20× and 40× (with 2× zoom) objectives. Scale bars in panel = 100 µm for 20× objective; 20 µm for 40× (with 2× zoom) objective. Data are representative of two independent experiments.

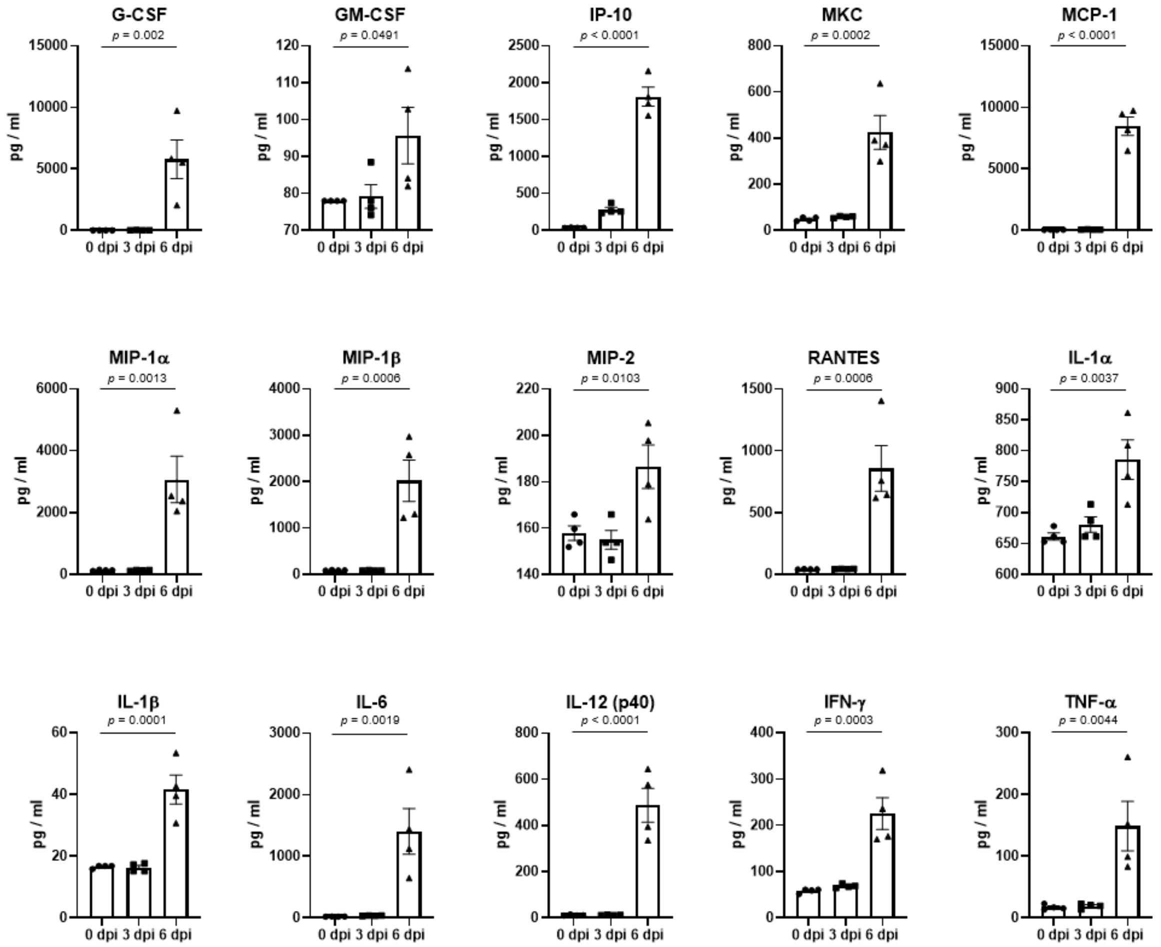

Supplementary Fig. 6. Multiplex cytokine analysis of the brain tissues of SARS-CoV-2-infected mice. The chemokine and cytokine levels of the brain were measured using multiplex immuno-analysis (n = 4 per indicated dpi). G-CSF, granulocyte-macrophage colony-stimulating factor; IP-10, C-X-C motif chemokine 10 (CXCL10); MKC, mouse keratinocyte-derived chemokine; MCP-1, monocyte chemoattractant protein-1 (CCL2); MIP, macrophage-inflammatory protein; RANTES, regulated upon activation, normal T cell expressed and presumably secreted (CCL5); Symbols represent means ± SEM. Statistically significant differences between the groups were determined using one-way ANOVA. SARS-CoV-2: severe acute respiratory syndrome coronavirus 2; dpi: days post-infection

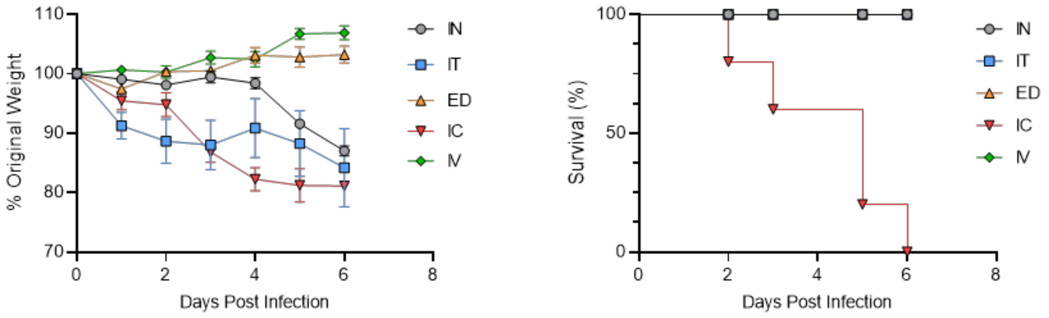

Supplementary Fig. 7. Body weight and survival rate following SARS-CoV-2 infection via various routes. K18-ACE2 mice were inoculated with 10^4 PFU SARS-CoV-2 via five different injection routes (n = 8 per injection route). Body weight (left) and survival (right) were monitored at the indicated dpi. Symbols represent means ± SEM. SARS-CoV-2: severe acute respiratory syndrome coronavirus 2; PFU: plaque-forming unit; dpi: days post-infection.

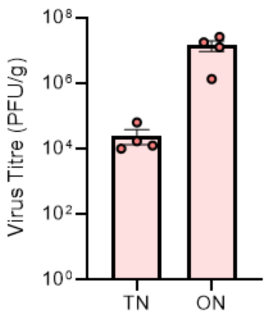

Supplementary Fig. 8. Viral loads in the trigeminal nerves and optic nerves of SARS-CoV-2-infected mice. Eight-week-old male K18-ACE2 mice were intranasally infected with 10^4 PFU of SARS-CoV-2 (n = 4). The infectious virus titre was analysed using plaque assay at 6 dpi. Symbols represent means ± SEM. TN: trigeminal nerves; ON: optic nerves.

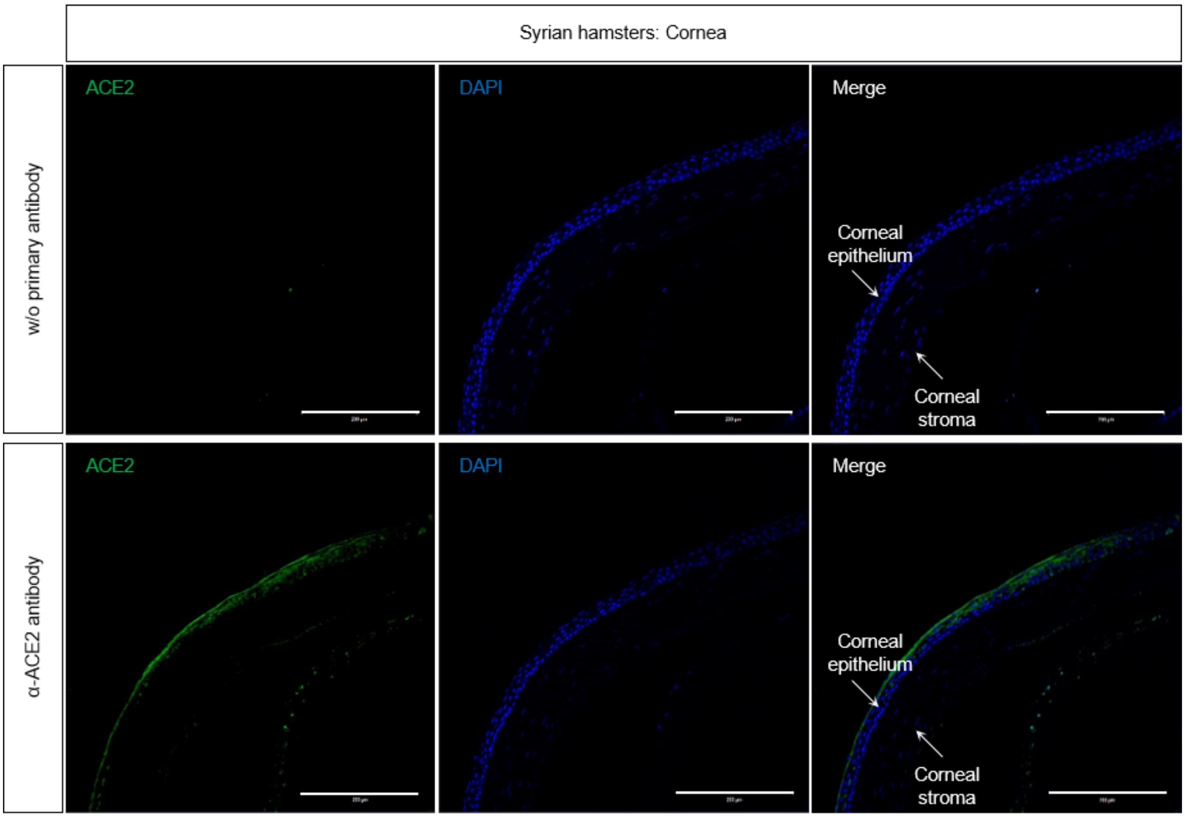

Supplementary Fig. 9. Representative confocal images showing immunofluorescence staining for ACE2 in the eyes of Syrian hamsters. Eye tissues of eleven-week-old female Syrian hamsters (*n* = 4) were collected and processed for immunofluorescence staining. Eye sections were stained for ACE2 with (lower) or without (upper) anti-human ACE2 primary antibody. DAPI staining (blue) was used to visualize the nuclei. Scale bars in panel = 200 µm.

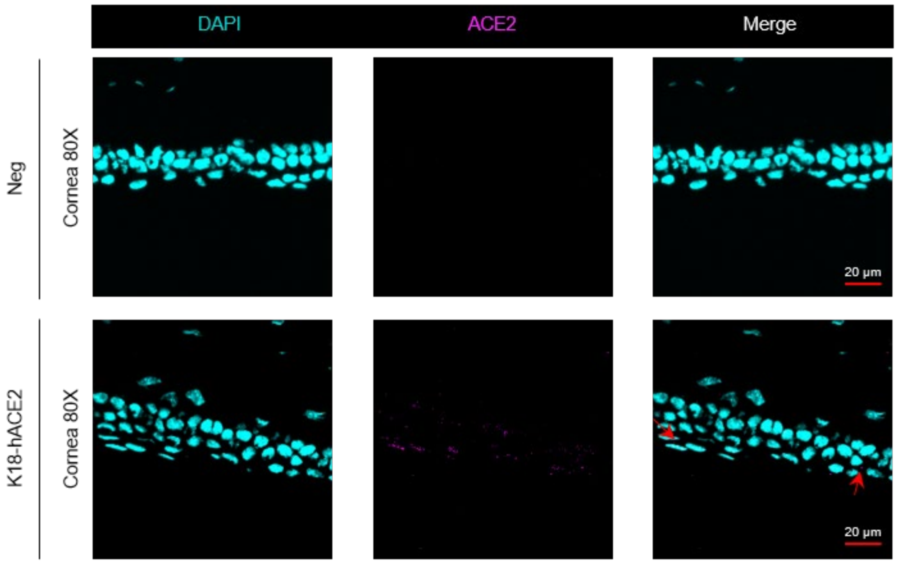

Supplementary Fig. 10. Representative confocal images showing immunofluorescence staining for ACE2 in the eyes of K18-hACE2 mice. Confocal microscopy of immunofluorescent staining of 10µm–cryosections of the eye of C57BL/6 (Neg) or K18-hACE2 mice. Images were acquired by confocal microscopy using a 40x (with 2x zoom) objective and are representative of n = 10. Scale bars in panel = 20 µm for 40x (with 2x zoom) objective. Data representative of two independent experiments.

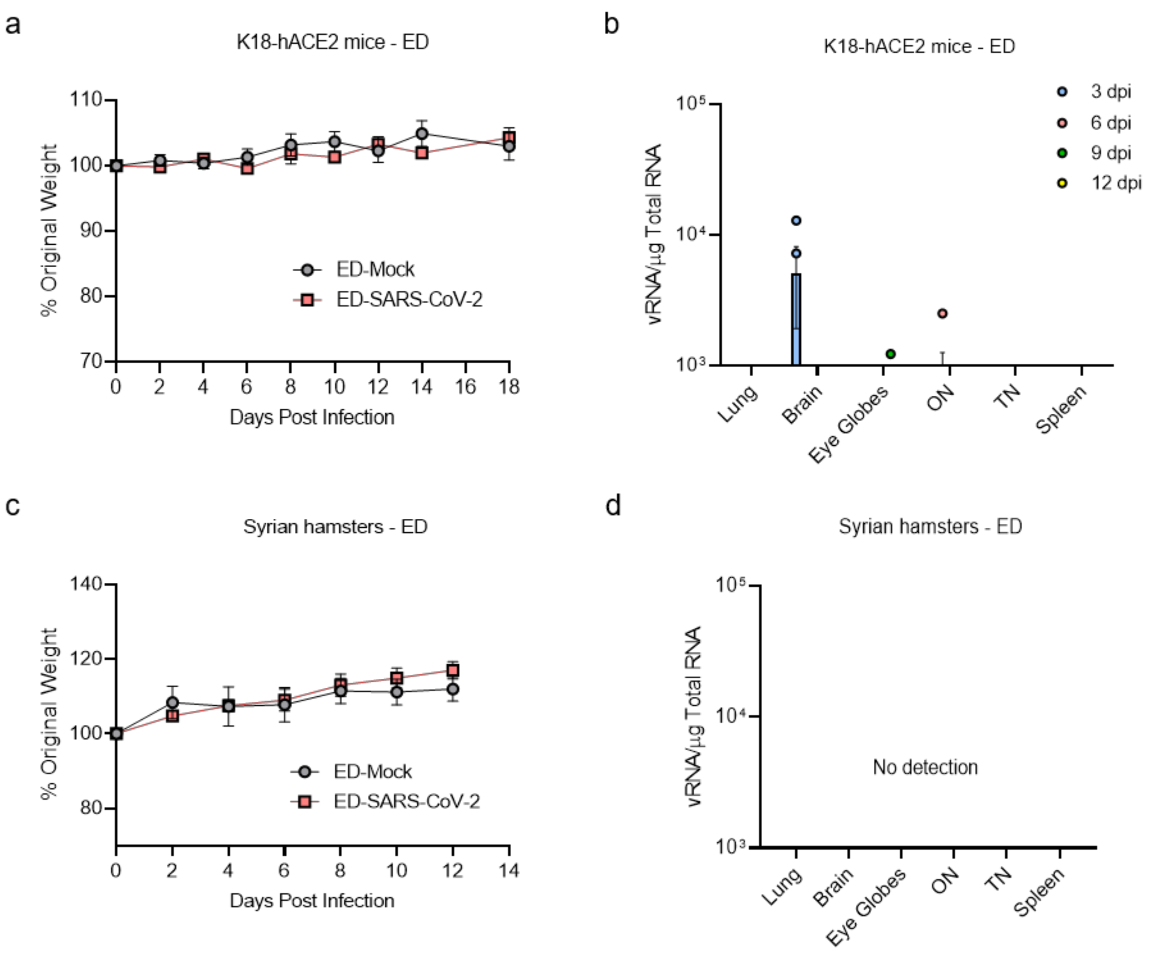

Supplementary Fig. 11. Body weight and viral loads in the lungs, brain, eye globes, trigeminal nerve, and optic nerve of K18-hACE2 mice and Syrian hamsters following SARS-CoV-2 infection via eye drop. K18-hACE2 mice (*n* = 16; *n* = 4 for 3, 6, 9, 12 dpi, respectively) and Syrian hamsters (*n* = 7 for 12 dpi) were inoculated with 10^4 PFU SARS-CoV-2 via eye drop. **a, c** Body weights of both animals were monitored at the indicated dpi. **b, d** Viral RNA levels in the lungs, brain, eye globes, trigeminal nerve, and optic nerve of mice were analysed at 3, 6, 9, and 12 dpi using RT-qPCR (**b**; 3 dpi, blue; 6 dpi, red; 9 dpi, green; 12 dpi, yellow), and those of hamsters were analysed at 12 dpi (**d**). Viral RNA copies were cut-off at 10^3 copies/µg. Symbols represent means ± SEM. SARS-CoV-2: severe acute respiratory syndrome coronavirus 2; PFU: plaque-forming unit; dpi: days post-infection
