# Supplementary figures and images for: Ocular tropism of SARS-CoV-2 in animal models with retinal inflammation via neuronal invasion following intranasal inoculation
Source: Nat Commun. 2022 Dec 12;13:7675. doi: 10.1038/s41467-022-35225-1 (PMC9743116; doi:10.1038/s41467-022-35225-1)

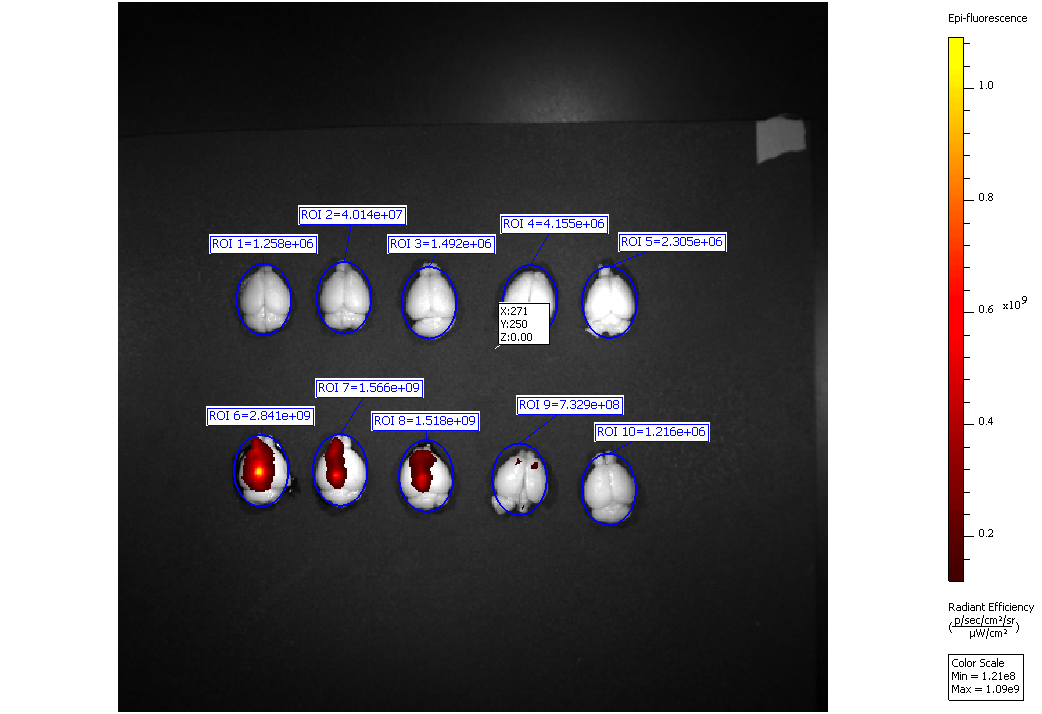

Supplement: Supplementary file 4 — Source Data [file 41467_2022_35225_MOESM4_ESM.zip › Figure 4d/Brain.tif]

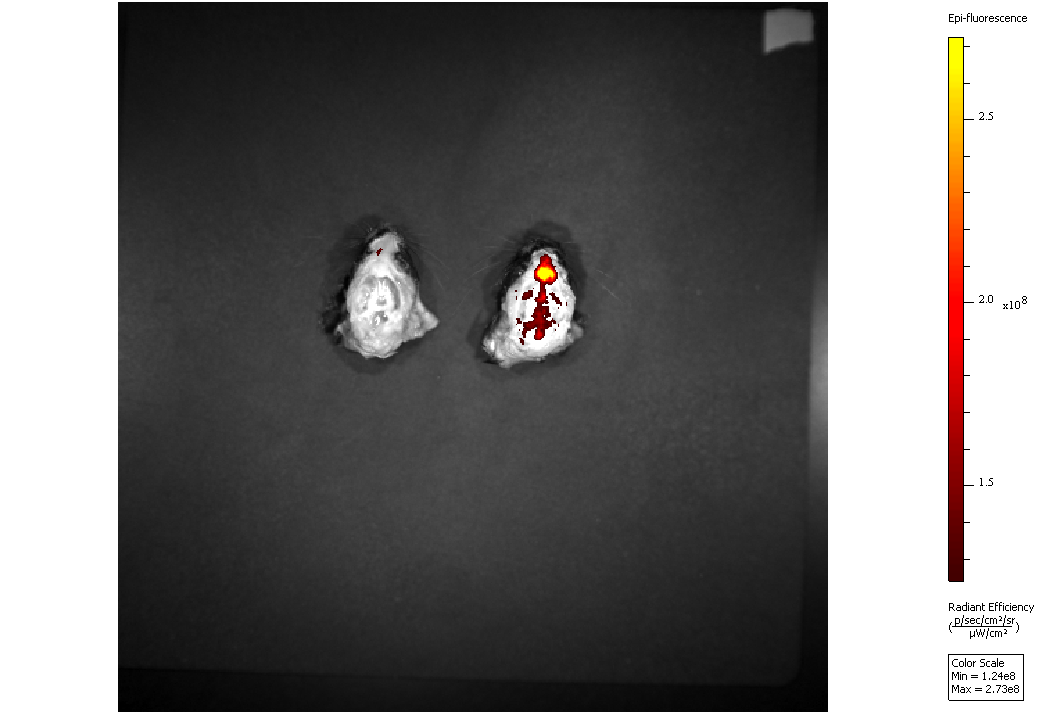

Supplement: Supplementary file 4 — Source Data [file 41467_2022_35225_MOESM4_ESM.zip › Figure 4d/Cranial cavity.tif]

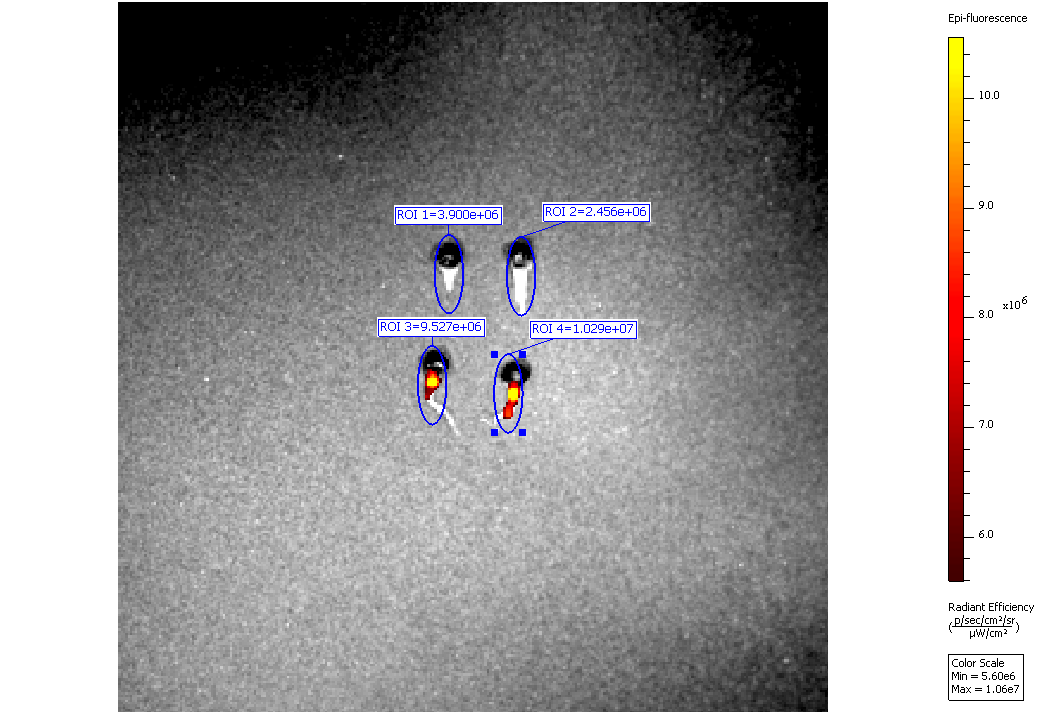

Supplement: Supplementary file 4 — Source Data [file 41467_2022_35225_MOESM4_ESM.zip › Figure 4d/Eyes.tif]

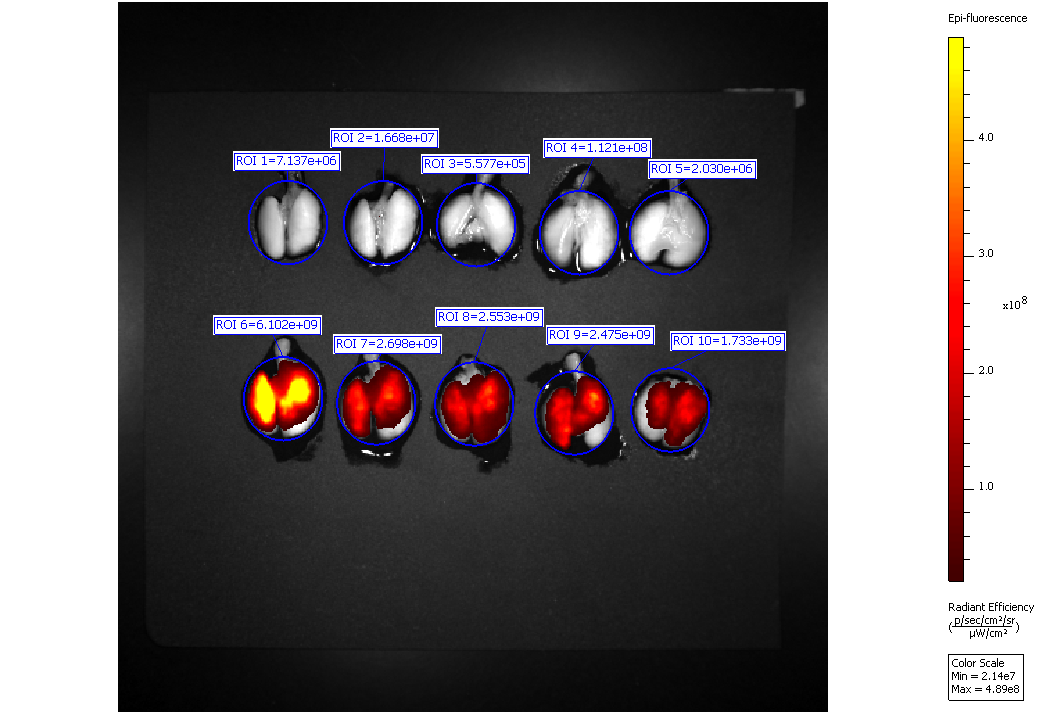

Supplement: Supplementary file 4 — Source Data [file 41467_2022_35225_MOESM4_ESM.zip › Figure 4d/Lung.tif]

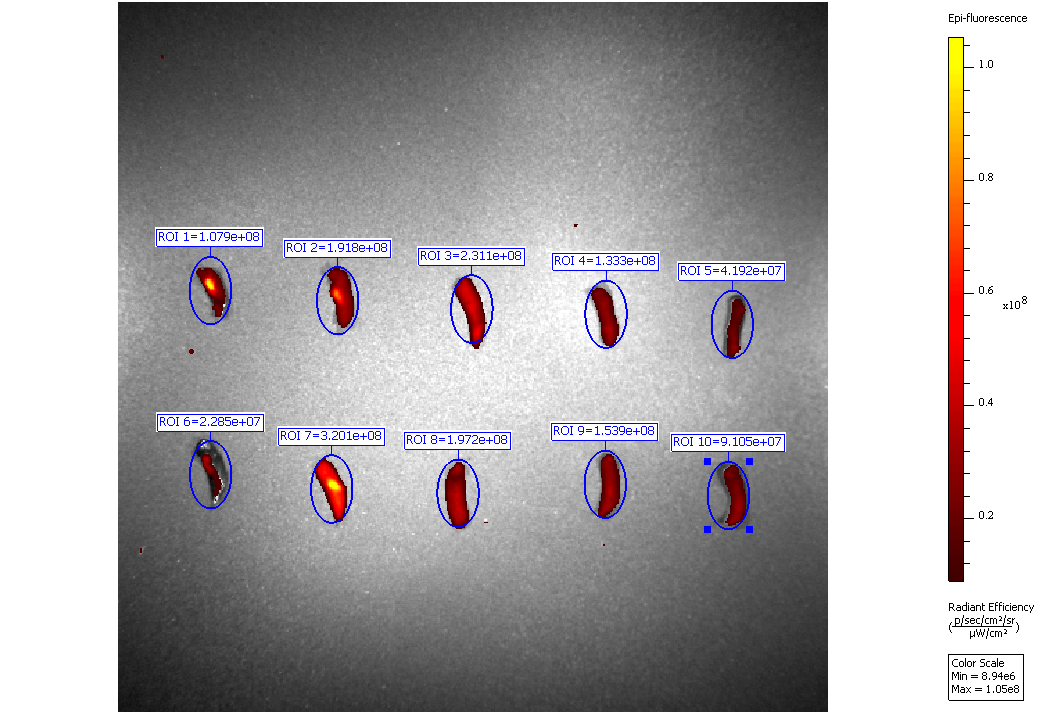

Supplement: Supplementary file 4 — Source Data [file 41467_2022_35225_MOESM4_ESM.zip › Figure 4d/Spleen.tif]
